# Changes in health after a work-related intervention among highly educated migrants in Norway: a pilot study

**DOI:** 10.1186/s12889-025-25025-9

**Published:** 2025-10-31

**Authors:** Khadra Yasien Ahmed, Yeneabeba Tilahun Sima, Astrid Lunde, Astrid Blystad, Wegdan Hasha, Lars T. Fadnes, Bernadette Kumar, Esperanza Diaz

**Affiliations:** 1 Department of Global Public Health and Primary Care, University of Bergen, Bergen, Norway; 2 Oral Health Center of Expertise in Western Norway, Bergen, Norway; 3 Department of Biomedicine, Faculty of Medicine, University of Bergen, Bergen, Norway; 4 Department of Addiction Medicine, Bergen Addiction Research, Haukeland University Hospital, Bergen, Norway; 5 Division for Health Services Research, Norwegian Institute of Public Health, Oslo, Norway

**Keywords:** Self-rated health, Migrants, Work-intervention, Integration, Mixed-methods, Pilot study

## Abstract

**Background:**

Highly educated individuals migrating to a new host country without work accreditation face various stressors that impact their health. Among them is the long waiting time for their integration as a resourceful workforce. In this study, we piloted a work-related intervention aimed at improving the health of highly educated migrants. We hypothesize that by including migrants in meaningful working-related activities, their self-rated health (SRH) and other health outcomes will improve.

**Methods:**

This is a non-randomized intervention pilot study examining the changes in participants’ health using an explanatory sequential design for evaluation. Baseline data was collected in Bergen for the intervention group and Kristiansand for the control group in 2023, with follow-up data collected after six months. The intervention consisted of working as assistant teachers at various health education programs at university level in Bergen for six months. Both groups answered a questionnaire that included SRH measured by a single validated item and other health measures. We calculated prevalence proportions and ratios, and differences in means using generalized estimating equations with 95% confidence intervals (CI), to estimate the changes in health outcomes adjusting for baseline confounders. Semi-structured interviews were conducted with the intervention group (*N* = 15) to gain deeper understanding of changes in health or other effects of the intervention.

**Results:**

Fifteen participants in the intervention and 62 in the control group completed both questionnaires. We found no changes between the groups in SRH, but significant improvements in general mental health measured with the crude General Health Questionnaire-12 (GHQ-12) -0.07 (-0.11;-0.03) and improved well-being measured with the adjusted World Health Organization-5 Well-Being Index (WHO-5) 0.09 (0.01;0.17) in the intervention group. Qualitative data indicated a positive intervention experience, explained by renewed self-confidence, family pride, improved stress management, empowerment and increased physical activity.

**Conclusion:**

Our pilot study suggested positive health changes from a work-related intervention in terms of improved general mental health and well-being. Quantitative and qualitative data were complementary. For confirmation of effects, this pilot study should be upscaled with a randomized trial design.

**Supplementary Information:**

The online version contains supplementary material available at 10.1186/s12889-025-25025-9.

## Introduction

To handle the current staff shortage in the health sector, Norway, a high-income country (HIC), will need more personnel and expertise [1, 2]. Despite this growing need, 42% of the migrants in Norway are overqualified for their jobs, and migrant health professionals make up a share of this number [3]. As the percentage of migrants increases, Norway will also become a more diverse nation, necessitating a more diverse workforce to meet the country’s future healthcare demands [4]. Research highlights the importance of minority health professionals in addressing health disparities as a vital link to their communities [5, 6]. Thus, utilizing the expertise of this professional group might benefit migrants as well as the host nation.

Migrants in high-income countries often face health risks from pre-migration stressors and post-migration challenges such as underemployment and unemployment [7]. Early experiences of exclusion or marginalization can have long-lasting effects on migrants’ social and economic integration in a new host country by slowing language acquisition and limiting engagement in civic life [8–11]. Language barriers, prolonged waiting times for accreditation by the regulatory agencies of the host nations [12, 13], and a lack of knowledge about the medical culture and healthcare system of the new country, may, moreover contribute to feelings of deskilling and difficulties with acculturation [14, 15]. These challenges restrict access to social capital, which is essential for successful integration [16].

Additionally, the absence of a professional network and assistance in navigating the bureaucratic system of the host nation can lead to dissatisfaction and poorer health outcomes by delaying integration and exclusion from working life [17, 18]. Research indicates that for many, the loss of both social and professional status undermines identity continuity and social recognition, which affects not only the individuals themselves but also their families [19, 20].

In addition to structural barriers, social experiences such as discrimination also shape health and integration outcomes. Integration and perceived discrimination are important determinants of health among migrants [21]. Even after controlling for socioeconomic status, research has indicated that migrants who experience discrimination report poorer health outcomes [22]. Discrimination can undermine the benefits of employment and integration by increasing psychological distress and reducing trust in institutions and the host society [23]. The integration paradox, which states that higher expectations and qualifications result in greater self-perceived discrimination when the expectations are not met, may particularly affect highly educated migrants [24]. This paradox may trigger stress and negatively impact both mental health and integration [25].

Unemployment has been repeatedly linked to poorer mental health, higher levels of stress, and lower self-rated health, especially for migrants whose qualifications do not match their current jobs [26, 27]. Long-term underemployment and status loss can trigger chronic stress responses, including allostatic overload, or the increase in the cumulative wear and tear on the body and brain brought on by prolonged or recurrent stress [28]. Allostatic load is associated with both anxiety and cardiovascular disease [29]. Moreover, long-term unemployment may result in social isolation and feelings of worthlessness, both of which are recognized risk factors for lower quality of life [30, 31].

Social isolation and a sense of meaninglessness are associated with both poor mental and physical health [32]. Furthermore, social isolation increases vulnerability to both anxiety and depression [33]. While unemployment and a lack of meaningful engagement are harmful to one’s mental and physical health, being employed, especially in positions that match one’s skills, can be health promoting [34, 35]. Structure, identity, and self-efficacy, all of which are protective elements for mental health, are enhanced by employment [36]. Additionally, employment may encourage participation in positive health-related behaviors including exercise and preventive care [37]. Social belonging, cultural adaptability, and recognition are all important aspects of integration in addition to employment [25]. These factors support integration by reducing exclusion and reinforcing a stable sense of identity [38]. Feeling accepted encourages migrants to form social and professional ties, which strengthens their integration process [39].

A persistent political and economic challenge in HICs has been the question of how to efficiently and sustainably integrate highly educated migrants into the workforce [15, 16] as workplace integration requires time [40, 41]. Concerns about the slow pace of inclusion in the labor market and civil society have led to a conceptual shift from a rights-based approach to one emphasizing individual responsibility for employment [42, 43]. These policy changes imply that labor market participation and personal activation are essential for acculturation into the new host society [44–46].

To improve the employability of highly educated migrants in their new host nation, studies worldwide attest to the importance of early interventions and assistance [12, 14, 17, 47, 48]. Mentorship, in which experienced professionals support and guide migrants during their transition into the workplace, is one of the elements of early interventions recommended for this group [48]. These early professional relationships have proven to be vital for re-integrating highly educated migrants into their former professions, and have simultaneously been found to promote health by reducing isolation [14, 49]. By reestablishing social connections and professional identity, mentorship and work-related interventions have been linked to lower stress, improved self-rated health, and mental well-being [50]. Work-related interventions may, moreover, improve mental health by restoring structure, meaning, and network [51]. They may also enhance self-efficacy by helping participants navigate complex systems such as accreditation and employment [14], while fostering a sense of belonging and integration, both key health determinants for migrants [52, 53], strengthening resilience and long-term well-being [38, 54].

Although acculturation to a new host country, meaningful work integration, and health status are all intimately connected and interact in a manner impacting every aspect of an individual’s life [22, 34, 55], the knowledge on ways to enhance the health and work integration of highly educated migrants in Norway is lacking. To address this void in knowledge, we have piloted a specifically designed intervention with the aim to improve the health and well-being of highly educated migrants with health educational backgrounds. We assessed if a work-related intervention where migrants are allocated to different tasks as assistant teachers in their respective health education fields could improve their health. We hypothesized that among highly educated migrants with health education backgrounds, participation in a work-related intervention will improve self-rated health (SRH), general mental health, well-being, and lastly, integration and discrimination scores, compared to a control group. This paper presents findings on the intervention’s health impact, while a feasibility assessment will be covered in an upcoming paper.

## Methods

### Study design, setting and data collection

**Fig. 1:**
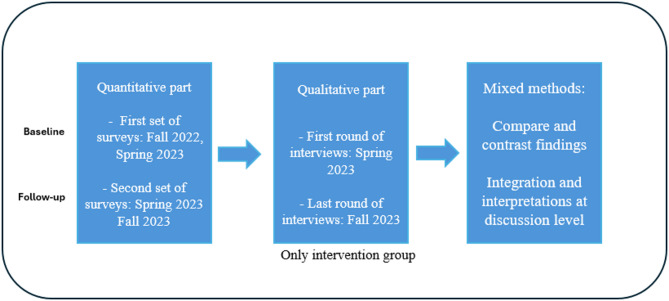
The explanatory sequential mixed methods study design (*N* = 123)

This is a non-randomized intervention pilot study examining the changes in participants’ health, using a sequential explanatory mixed methods design [56]. Pilot studies can be valuable for assessing feasibility and fidelity, finding mitigation measures, and providing lessons for future full-scale implementations [57]. The analysis of the quantitative and the qualitative material was conducted separately as described by Creswell and Clark [58] (Fig. 1). Over six months, we followed a group of highly educated migrants in Bergen alongside a group based in Kristiansand, Norway. We will use the terms intervention group and control group in the paper. A fully detailed description of the methods for the intervention has been presented elsewhere [59].

### The intervention

Three days of preparation preceded the intervention, during which the participants were introduced to the study, met other participants, and discussed potential problems and solutions to these. The intervention group attended one of two academic institutions in Bergen for a minimum of two days a week for six months. Participants were matched with mentors with health backgrounds as close to themselves as possible, serving as the main sources of support at the placement site. Throughout the six-month intervention period, the participants conducted tasks such as teaching, research, observation of mentors, and laboratory work. Participants had varying levels of Norwegian proficiency (minimum A2), and while this posed some initial challenges at the intervention sites, support from mentors and daily exposure to Norwegian in academic settings facilitated language development.

### Inclusion and exclusion criteria

Migrants living in Bergen with a health education gained abroad, but lacking full Norwegian accreditation to work and language proficiency of A2-level or higher (pre-intermediate), were recruited as explained elsewhere [60]. The control group was recruited in Kristiansand from the introductory program for newly arrived migrants, with minimum high school education, A2-level language proficiency, but no health education requirement. The exclusion criteria for the intervention group was mental health problems. We used a mean score of >1.85 measured with Hopkins Symptom Check List (HSCL-10) as a screening for such likely problems followed by a consultation with a psychologist when needed. No one was excluded on this ground. There were no exclusion criteria for the control group, as they were not partaking in the intervention.

### The study population

**Fig. 2:**
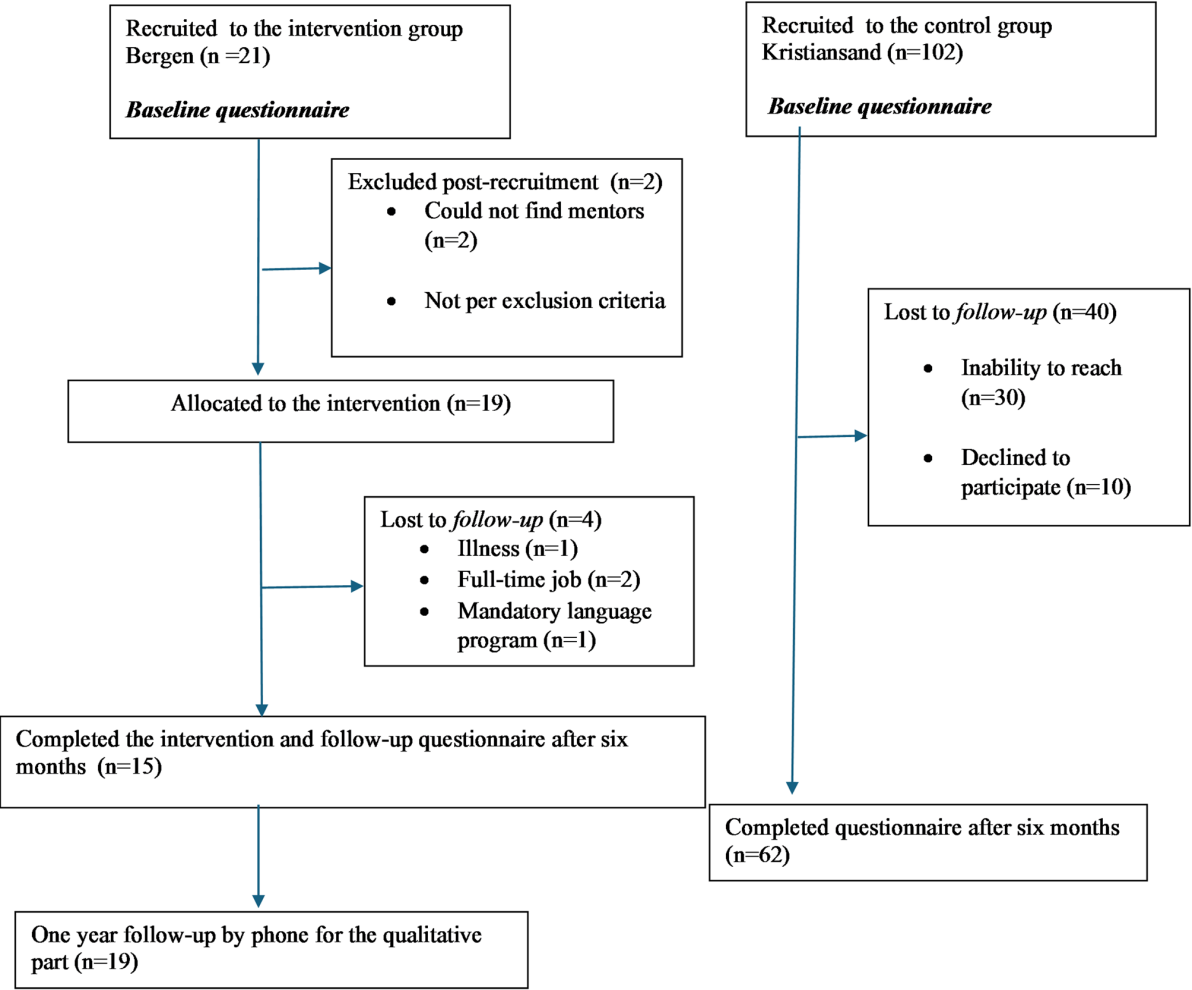
Flowchart of the study population (*N* = 123)

A total of 21 participants (13 women and 8 men) were recruited to the intervention group and 102 participants (41 men and 61 women) to the control group (Fig. 2). Baseline measures were collected through a self-administered survey during the fall of 2022 and 2023. 19 were allocated to the intervention group, 4 were lost to follow-up and 15 participants completed it. Nine were medical doctors, three pharmacists, one dentist, one nurse, and one radiographer. In the control group, we included 102 participants, of whom 62 completed both surveys. Sociodemographic information for both groups is shown in Table 1.

**Table 1:** Sociodemographic and migration related factors in the intervention group and control group (*N* = 123)

	Intervention (*n* = 21) *n* (%)	Control (*n* = 102) *n* (%)
Age groups		
20–29	3 (14)	33 (32)
30–39	11 (52)	41 (40)
40+	7 (33)	28 (27)
Gender		
Women	13 (62)	61 (60)
Marital status		
Married/cohabitating	17 (81)	73 (72)
Single/divorced/widowed	4 (19)	28(28)
* Missing*	0	2(2)
Emigration reason		
Refuge/family reunification	15 (71)	86 (84)
Work or studies	6 (29)	16 (16)
Continent of birth		
North and South America	1 (5)	6 (6)
Asia	14 (63)	61 (60)
Europe	3 (16)	17 (17)
Africa	3 (16)	10 (10)
* Missing*	0	8 (8)
Year of arrival		
2016–2020	14 (67)	32 (31)
2021–2023	7 (33)	70 (69)
Number of children		
0	5 (24)	48 (47)
1	5 (24)	26 (25)
2+	11 (52)	28 (27)
Educational level		
Vocational education	0 (0)	13 (13)
Minor degree	0 (0)	10 (11)
Bachelor’s degree	3 (14)	42 (41)
Master’s degree, integrated 6-year program or PhD	18 (86)	35 (34)
* Missing*	0	2 (2)
Academic discipline		
Natural sciences and technical subjects	0 (0)	35 (34)
Health sciences	21 (100)	14 (14)
Other	0 (0)	53 (52)

### Quantitative measures

The questionnaire built upon questions on sociodemographic and migration factors from the Changing Health and Health Care Needs Along the Syrian Refugees’ Trajectories to Norway (CHART)-study [61]. It was available in both Norwegian and English. Before and after each round of the intervention, participants in both Bergen and Kristiansand completed a survey. The intervention was implemented in two consecutive rounds, each involving a distinct group of participants. For the first round, baseline data were collected in November 2022, with follow-up data in June 2023. For the second round, a new group of participants were recruited, with baseline data collected in June 2023 and follow-up in December 2023.

### Independent variables

The sociodemographic variables are thoroughly described in the protocol and shown in Table 1. For sensitivity analysis, educational discipline was dichotomized as either health science or other educational sciences.

### Dependent variables

Our main health outcome was SRH measured by a single-item question: “How do you consider your health now?”. This was answered using a five-point scale from very poor to very good [62, 63]. For analysis, responses were grouped into “very good” and “not very good” (including poor, neither, very poor, and good). Higher scores indicate better self-perceived health status.

General mental health was evaluated using the General Health Questionnaire (GHQ-12) [51], where higher scores reflect poorer mental health. The HSCL-10 [64] was utilized to assess symptoms of anxiety and depression and higher scores indicate greater psychological distress. Scores ≥ 1.85 indicate clinically significant mental distress [64]. Well-being was measured through the Well-Being Index (WHO-5) [65], where higher percentage scores denote better well-being.

Other secondary outcomes were assessed using the Sense of Coherence Scale (SOC-13) [66], with higher scores representing a stronger sense of coherence. Integration was assessed using the Immigration Policy Lab Integration Index (IPL-12) [67], higher scores indicate better integration across linguistic, social, and psychological domains. Lastly, we included questions about self-perceived discrimination using the European Social Survey 2010 [68], higher scores indicate greater self-perceived discrimination. Scores from each measure were summed up and standardized from 0 to 1, where 1 indicates a higher score.

### Statistical analysis

Sociodemographic- and migration-related variables measured at baseline were described for both the intervention and control groups. Continuous variables were described using mean and standard deviation (SD), while categorical variables were presented as counts and proportions [69].

We used generalized estimating equations (GEE) to analyze changes in outcomes over time, accounting for repeated measures within the same subject. In line with the modified intention-to-treat principle, we included all available baseline data and any follow-up data in the model. The longitudinal data were organized in a long format, each individual having either one (baseline) or two data points (baseline and follow-up). We did both crude and adjusted analysis adjusting for age, gender, year of arrival, and number of children. For the only dichotomized outcome, SRH, we used a log-link function and binomial distribution, reporting results as prevalence proportions and ratios with 95% confidence intervals (CI). For the dichotomized outcome, we used age as a continuous variable. Continuous outcomes were analyzed using an identity link and Gaussian distribution, presenting regression coefficients (means and differences in means) and their CI. Age was used as a categorical variable in the continuous outcomes. To evaluate the intervention effect, we included an interaction term between time and group. The potential intervention gains were estimated, comparing the intervention with the control group as a reference. The statistical significance threshold alpha was set at 0.05. We analyzed the data using STATA/SE software version 18.1 (StataCorp LLC, Texas, USA).

### Sensitivity analyses

A total of 46 individuals did not complete the follow-up measurement, which could potentially influence our estimates. The GEE analysis we performed assumes that data are missing completely at random (MCAR). To address this, we conducted random effects analyses (mixed model), comparing the estimates from the GEE analysis to the estimates from the similar mixed model analysis, assuming the missing data occurred at random (MAR) [70, 71] (Supplementary data). We also performed inverse probability weighting (IPW) combined with GEE to adjust for potential bias due to missing outcome data [72]. We examined baseline covariates among participants with and without missing follow-up data. The distribution of variables such as number of children and year of arrival differed by missingness status, suggesting that the data are more likely Missing at Random (MAR) rather than MCAR. Distribution of missing data can be found in the Supplementary.

All the participants in the intervention group had a health science educational background. To determine if our findings could be attributed to this factor, we conducted stratified analysis in Kristiansand, categorizing participants by their educational background (health versus non-health) and comparing the results between these groups (data not shown).

### Qualitative measures

Semi-structured interviews were conducted at the end of the intervention among participants in the intervention group only (*n* = 15). The interviews lasted between 20 and 30 min. The audio recorded interviews were transcribed and analyzed drawing upon the six-step reflexive thematic analysis framework by Braun and Clark [73]. Data were analyzed with an inductive-deductive approach to explore themes under the pre-defined areas of health and other emerging themes [74]. The interpretation of the codes and themes were discussed among the co-authors ED, KYA and AB. One year after the intervention, we followed up the intervention group by phone to get feedback reading self-perceived effect on health and reflections on participation in the intervention.

### Ethics

The study was registered at the National Center for Research Data (NSD/SIKT) (reference number 624616) and is conducted in line with the Helsinki Declaration. Written informed consent was obtained from all participants in the quantitative part, and oral consent was obtained for the qualitative interviews. The Regional Committee for Medical Research Ethics Western Norway (REK West) assessed this project to be outside their scope and hence the need for approval was waived (reference number 480807). Quotations from the participants are assigned pseudonyms.

## Results

In Bergen, 15 of 21 participants completed the intervention and both assessments, while 62 of 102 participants in Kristiansand completed both assessments, resulting in attrition rates of 29% and 39% respectively.

While both groups shared similarities in sociodemographic characteristics such as gender and marital status, they differed in others, including educational level, time of residence in Norway, and number of children (Table 1).

**Fig. 3:**
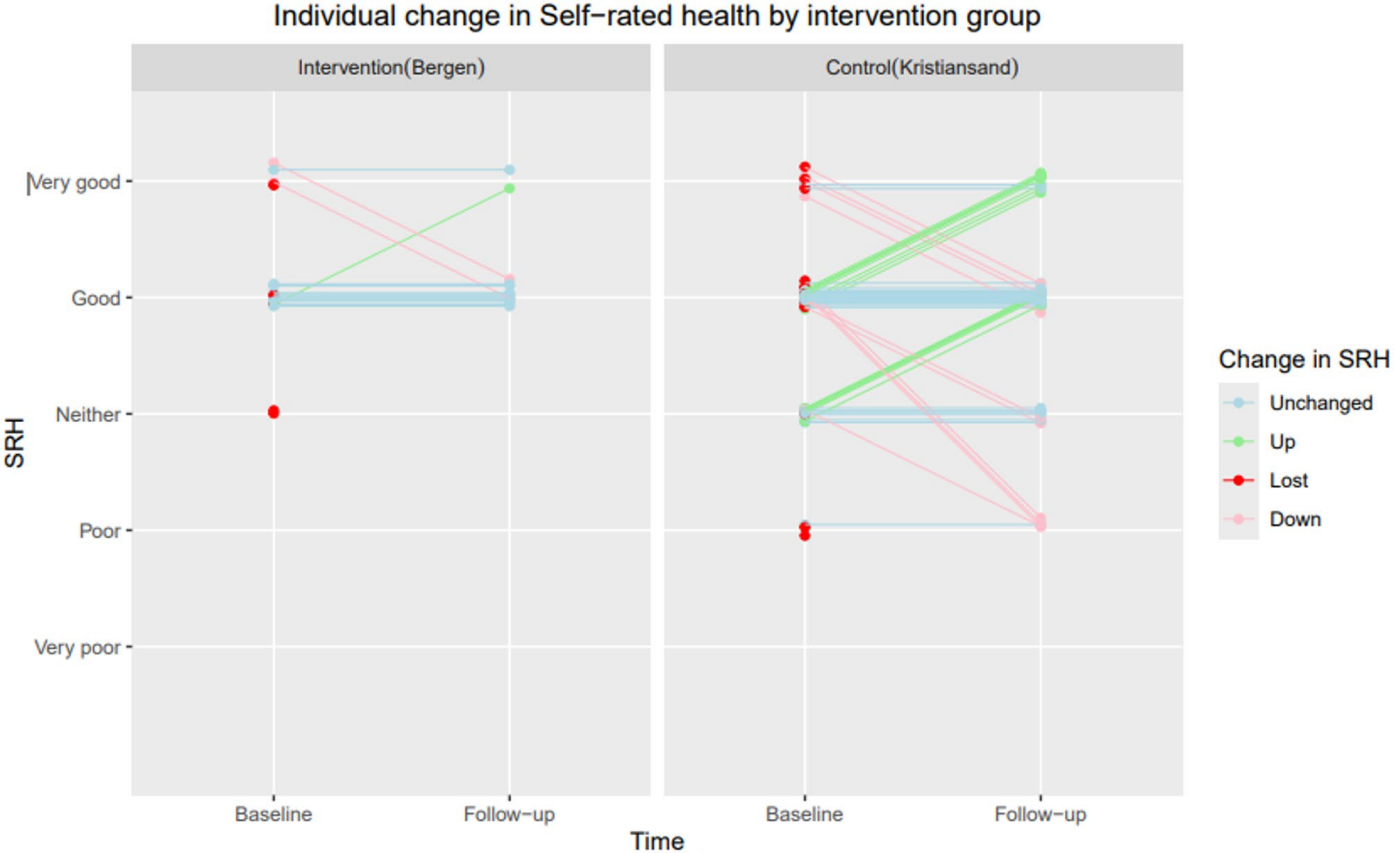
The changes in SRH for each individual from baseline to follow-up for intervention and control group (*N *= 123)

Figure 3 illustrates the changes in SRH for each individual from baseline to follow-up (N=123). At baseline, 76% (16/21) and 89% (91/102) of the participants in the intervention and the control group respectively reported good SRH. In the intervention group, the majority maintained the same level of good health from baseline to follow-up. In the control group, changes in SRH were more varied. Loss to follow-up was balanced between the two groups with respect to SRH, as illustrated by the red dots in Figure 3 below.

Table 2 presents the changes in health and non-health -health outcomes in the intervention and control group from baseline to follow-up. Following the six-month intervention period, our findings suggest improvement in well-being 0.09 (0.01, 0.17) (adjusted) and a reduction in mental distress measured by the GHQ-12 −0.07 (−0.11, −0.03) (crude) in the intervention group compared to the control group. However, no significant differences were observed between the groups in SRH, anxiety/depression or sense of coherence. As for the sensitivity analysis, the change in SRH and other outcomes did not differ by educational groups among participants in Kristiansand (results not shown). Results from the random effect analyses were comparable with GEE analyses (Supplementary file).

**Table 2:** Changes in self-rated health (SRH), other health outcomes, integration and discrimination from baseline to follow-up and effect of the intervention (N* = 123)*

Outcomes at baseline and follow-up in intervention and control groups				Effect in intervention versus control groups		
	n (%)	n (%)	n (%)	n (%)	Crude RPR ^a^ (95% CI)	Adjusted RPR ^a,c^ (95% CI)
SRH (Very good)	5 (23.8%)	2 (13.3%)	11 (10.8%)	11 (17.7%)	0.35 (0.08, 1.66)	0.35 (0.08, 1.62)
	Mean (SD)	Mean (SD)	Mean (SD)	Mean (SD)	Crude difference ^b^ (95% CI)	Adjusted difference ^c^ (95% CI)
General Health Questionnaire-12 (GHQ-12)	0.48 (0.01)	0.45 (0.01)	0.44 (0.08)	0.49 (0.01)	−0.07 (−0.11, −0.03)	−0.07 (−0.11, −0.02)
Hopkins Symptoms Checklist-10 (HSC-10)	0.28 (0.04)	0.25 (0.03)	0.28 (0.01)	0.21 (0.01)	0.03 (−0.07, 0.12)	0.02 (−0.07, 0.11)
Well-being Index-5	0.67 (0.04)	0.78 (0.02)	0.67 (0.01)	0.69 (0.02)	0.09 (−0.01, 0.19)	0.09 (0.01, 0.17)
Sense of coherence-13	0.55 (0.01)	0.55 (0.02)	0.55 (0.01)	0.55 (0.01)	0.01 (−0.07,0.08)	0.01 (−0.07, 0.08)
The Immigrant Integration Index − 12	0.56 (0.21)	0.65 (0.23)	0.62 (0.01)	0.67 (0.01)	0.04 (−0.02, 0.10)	0.07 (−0.02, 0.17)
Discrimination	0.22 (0.04)	0.15 (0.04)	0.10 (0.01)	0.13 (0.01)	−0.10 (−0.21, 0.02)	−0.10 (−0.21, 0.02)

^a^*RPR* Ratio of prevalence ratio: prevalence ratio in the intervention group divided by the prevalence ratio in the control group (reference)

^b^ Difference: differences in differences in means

^c^ Adjusted for gender, age, year since arrival and number of children

### Qualitative data

There was a substantial degree of coherence in the findings across the qualitative interviews, and although the participants expressed their experiences in diverse ways, major themes were repeated across the 15 interviews among the participants that completed the intervention. Most of the participants shared similar experiences on how work-related intervention improved their self-confidence and mental health. The four participants who withdrew from the intervention also reported similar experiences of improved health and satisfaction with the intervention during the follow-up interview one year post-intervention. The main themes that emerged were renewed self-confidence, family pride, improved stress management, empowerment and physical activity. An overview of the themes and codes identified can be found in the Supplementary file.

### Renewed self-confidence and empowerment through professional reintegration

Numerous participants reported that they during the intervention learned more about the Norwegian health system, including how to apply for their health licenses. This was linked to a sense of relief because they had spent much time and energy trying to comprehend the system alone without success:

*“I know the steps now*,* and I know that I can talk to people that can help me. Now I have a plan. I’m sure many others in the project do too. “(Maryam).*

Several participants talked about how their confidence increased post-intervention:

*“When you’re in the process of beginning everything*,* you feel like you’re starting everything from zero*,* but this was like jumping to 50%. I felt like myself again. Just being able to be present in a workplace. It gives you confidence and empowerment.” (Thabita).*

The participants used numerous occasions to reflect on their long journeys, reaching a momentum at last: *“I felt that I was in the right place. It was not like when I first came and was hit by a wall” (Badr).*

All the participants expressed the challenges they faced in Norway in being prevented from utilizing their knowledge and experiences from their former professions. For many, the belief in ever re-entering their professions had seemed far-fetched. Not working was also linked to the experience of deteriorating mental health. However, amid uncertainty and knowledge of the long and uncertain accreditation process, many retained a strong hope of one day being able to practice their professions again. One participant shared how her first day of placement at a hospital after an extended period of waiting increased her pride in herself:

*“It was a huge accomplishment to just walk through the hospital and sit in their meetings and hear about the patients. I felt like I could use my expertise and my abilities.” (Hilal).*

However, for some, the joy of being included in their previous profession simultaneously brought up what seemed to be a sense of confusion. After a long time of waiting, having to accept that they were in fact a part of a professional group was difficult, as many had lost belief in ever rejoining their profession in Norway.

*“I feel my health improved somehow being in my own environment*,* but I am sad too. I like the memories I have from here. Now I feel I’m a bit more like somebody. Although I was kind of comfortable with being nobody. I have a mix of inspiration*,* but also some feeling of lostness” (Ali).*

### Family pride and wellbeing

Many reported how also their families took pride in seeing them enter work. Though expressed by both men and women, women in particular experienced that by having a job, their children saw them in a new light. “*After I started with this program my children were also very proud. They say my mother works at the university because of my access card to the university.” (Yamina).*

Several women reported that the perception their children had of them particularly changed as they moved from a status of stay-at-home mothers to working mothers:

“*Honestly*,* my family was affected a lot. My children say*,* “Mum has a job”*,* they see I’m going and doing something. It’s not like before when I was sitting at home.” (*Kawther).

### Improved stress management through meaningful activities

According to many of the informants, their mental state improved during the intervention. Many stated that they experienced symptoms of depression, stress and uncertainty prior to the intervention due to joblessness and long waits for authorization. Work acted as a buffer against stress: *“I think going to work helped me very much mentally because I entered my former professional environment. I was able to create a network*,* and I learned. I think that gradually my stress lowered.” (Rashid).*

However, a few participants experienced that their health did not change much. One participant stated that he already had good health to begin with and responded: “My *health did not change.” (Omar).*

### Increase in physical activity

For many, participation in the intervention also meant more physically active tasks and less monotonic and sedentary activities. A distinction between men and women’ experiences was noted with few men reporting more physical activity during the intervention.

*“Physically*,* I was out every day and walked. We walked every day from the hospital to the university and met outside to eat together. Before I used to eat at home*,* and I didn’t know what I was going to do*,* but now I go out and learn*,* every day.” (Fatima).*

## Discussion

Using both quantitative and qualitative methods, this pilot study suggests that there were improvements in health after participation in a work-related intervention among highly educated migrants.

While there were no differences in changes in SRH between the intervention and control group, other health outcomes seem to be more sensitive to changes in health in this population, like well-being and general mental health. The intervention did not have the anticipated effect on SRH. No significant improvement in SRH could be due to a real lack of effect, but also to the already high SRH among participants at baseline, which was comparable to the general Norwegian population [75]. While studies typically categorize SRH as poor vs. good [76–78], we used “not very good” vs. “very good” because most participants reported good SRH at baseline, which may explain why we did not observe changes in SRH between the groups.

To evaluate the effect of the intervention on mental health, we assessed changes using the GHQ-12 and HSCL-10 scales. While a significant improvement was observed in the GHQ-12 scores, no changes were observed in the HSCL-10. This discrepancy may be due to the GHQ-12’s focus on everyday stressors, which could have been more directly impacted by the intervention [79]. As suggested by the qualitative interviews, stress levels among participants prior to the intervention were high due to unemployment and other stressors, and GHQ-12 scores were similarly high prior to the intervention. In alignment with this finding, two interventions examining pain reduction through group physiotherapy and improvement in mental health using Teaching Recovery Techniques among Syrian refugees also failed to find significant results when using SRH as an outcome, while obtaining significant improvements measured by GHQ-12 [80, 81]. Improvements in GHQ-12 scores were also observed among migrants participating in a five-week health promotion program in Sweden [51]. Our qualitative findings also suggest an improved ability to manage stress among the participants following the intervention. Finding easier ways to apply for authorization due to their new networks also lessened stress levels. The participants’ internal resilience and capacity to adapt to challenges in a new host country may also have accounted for improvements in stress management and mental health [35].

The intervention appeared to have a positive role on participants’ well-being, such as social inclusion and professional reintegration. A study from Denmark suggests that labour market participation can enhance the cultural assimilation of migrants, specifically when there is interaction with non-migrants at the workplace [82]. Interaction with Norwegians at the institutions was particularly important for supporting the development of Norwegian language proficiency among the participants, despite initial language barriers and varying levels of fluency. Inclusion at work and educational programs have also all been associated with improved well-being among migrants [12, 14]. The improved adjusted WHO-5 scores were also in line with the qualitative data, where participants reported improved well-being due to meaningful work-related activities. Migrants participating in a work intervention in Germany also showed improved WHO-5 scores. These results were attributed to a structured intervention and social assistance for the participants [83]. Consistent with our findings, qualitative evaluations of comparable programs targeting highly educated migrants highlighted the significance of professional engagement and mentorships for the migrants’ improved self-confidence and language acquisition [14, 84, 85].

Although our pilot did not have power to analyze the results by gender, there were indications of differences in effect related to gender, as women more often reported improvement in their physical health during the intervention period and experienced being acknowledged by their children for their new job situation during the intervention period. This should be further explored when upscaling this intervention.

Perceived discrimination has been associated with poor health outcomes among migrants in Norway [86, 87]. The higher levels of reported discrimination at baseline in the intervention group may be due to the integration paradox, as their education did not translate into the same socioeconomic status as the non-migrant population with comparable educational backgrounds [24, 88]. In our study, discrimination scores were reduced in the intervention group, as reflected by a reduction in mean score, while Kristiansand showed a slight increase in mean score. Although the adjusted difference − 0.10 (−0.21; 0.02) was not statistically significant, the trend suggests a potential positive effect of the intervention on perceived discrimination. These improvements may be attributed to enhanced language proficiency, better understanding of the host country’s systems, and increased interaction with the majority population post-intervention [89]. Improvements in integration could also be supported by an increased sense of well-being, improved self-confidence, and less mental stress [90]. In line with existing literature, our findings also imply that there may be a bidirectional relationship between improvements in integration and improvements in well-being [25, 91].

### Strengths and limitations

Our study has several strengths. First, to the best of our knowledge, this is the first study to pilot an intervention for highly educated migrants with a main outcome of health improvements in Norway. We included a wide variety of health professionals and used quantitative and qualitative methods to evaluate the effect of the intervention. The follow-up interviews conducted one year after the intervention added depth by capturing early post-intervention health outcomes. Lastly, this intervention highlights the importance of intersectoral cooperation in meeting future integration demands.

However, it also has limitations. The intervention could not be either randomized or blinded. As a pilot, we had a limited sample size. Furthermore, the studies’ reliance on self-reported data is a limitation, as it could be prone to recall bias. The inclusion criteria in each site were different, making the intervention and control groups inherently different, which challenges comparability. While we adjusted for key sociodemographic variables and stratified by education, residual confounding from other unmeasured factors cannot be ruled out. The multiple roles of the first author could have posed a challenge in terms of potential socially desirable reporting. We did not conduct qualitative interviews in Kristiansand, which excluded this group’s integration experience. This paper does not include the mentors’ perspectives nor a feasibility assessment. As we lost quantitative follow-up data from four participants, we attempted to partially mitigate this by conducting a 12-month qualitative follow-up with all participants, including those who discontinued the intervention, to capture their experiences and self-perceived health effects.

Although designed for health-educated migrants, the intervention’s focus on psychosocial factors like self-esteem and self-efficacy as linked to work participation suggests potential relevance also for other skilled migrant groups. Self-esteem and self-efficacy mechanisms are broadly linked to improved health and integration outcomes [92, 93], which may support a higher degree of generalizability. However, sector-specific differences may limit transferability. Future studies should explore similar approaches in non-health migrant professionals.

### Future lessons

This pilot offers important lessons. GHQ-12 and WHO-5 might be better tools than SRH to detect changes in health in this group of migrant professionals. In addition, measuring physical activity level pre- and post-intervention is recommended. Differential effects by gender should also be further explored. It is advised to have as similar a control and intervention group as possible or use a waiting list design. To avoid social desirability bias in the future, a blinded outcome assessment whenever feasible is recommended.

## Conclusion

Despite a small sample size, the results from the pilot intervention indicate positive improvements in health-related outcomes—particularly in general mental health and well-being. The intervention appears to have had a beneficial effect, as supported by the qualitative data. Moreover, this pilot intervention suggested that academic environments could facilitate the integration of highly educated migrants, accelerating their acculturation in a new host country. Our research adds to the evidence base that is required to plan and execute interventions for highly educated migrants. However, due to the limited sample size and potential influence of unmeasured confounders, our findings should be interpreted with caution. To verify potential effects, our intervention should be scaled up with trial design and sufficient power.

## Supplementary Information

Supplementary Material 1.

Supplementary Material 2.

Supplementary Material 3.

Supplementary Material 4.

## Data Availability

The datasets analyzed during the current study are not publicly available due to the privacy of the participants but are available from the corresponding author on reasonable request.

## Abbreviations

CHART: Changing Health and Health Care Needs Along the Syrian Refugees’ Trajectories to Norway
CI: Confidence intervals
GEE: Generalized estimating equations
GHQ-12: General Health Questionnaire-12
HIC: High-income country
HSCL-10: Hopkins Symptom Checklist-10
IPL-12: The Immigration Policy Lab Integration Index
IPW: Inverse probability weighting
NSD/SIKT: National Center for Research Data
SD: Standard deviation
SOC-13: Sense of Coherence Scale-13
SRH: Self-rated health
TRT: Teaching Recovery Techniques
WHO-5: World Health Organization-5 Well-Being Index
