# Supplementary material for: Changes in health after a work-related intervention among highly educated migrants in Norway: a pilot study
Source: BMC Public Health. 2025 Oct 31;25:3699. doi: 10.1186/s12889-025-25025-9 (PMC12577434; doi:10.1186/s12889-025-25025-9)
Supplement: Supplementary file 1 — Supplementary Material 1. [file 12889_2025_25025_MOESM1_ESM.pdf]

UNIVERSITY OF BERGEN

Samtykke- og invitasjonsbrev til deltagelse i studien:

"Helse gjennom meningsfulle integrerende yrkesrettede aktiviteter for høyt utdannede innvandrere- en pilot intervensjonsstudie"

Bakgrunnsinformasjon: formål med studien

Universitetet i Bergen gjennomfører en pilot-intervensjonsstudie som skal tilrettelegge for at høyt utdannede migranter med helsefaglig bakgrunn som ikke er godkjent i Norge, kan delta i utdanningsrettede aktiviteter ved UiB og HVL. Pilotstudien er del av en PhD-avhandling.

Intervensjonen skal måle effekten av meningsfull integrering på helse blant migranter og deres familier. Med meningsfull mener vi at deltakerne skal bruke så mye av kompetansen deres som mulig. Videre vil vi også undersøke hvordan migrantenes tilstedeværelse ved utdanningsinstitusjonene bidrar til økt kulturell bevissthet og kulturell kompetanse blant ansatte og studenter.

Resultatene av forskningen skal bidra til økt kunnskapsgrunnlag om meningsfull integrering blant høyt utdannede migranter i Norge. Dette er et invitasjonsbrev til å delta i studien og samtykke for å gjennomføre spørreundersøkelsene to ganger.

Hvem er ansvarlig for forskningsprosjektet?

Prosjektleder er Dr. Esperanza Diaz. Phd-student Khadra Yasien Ahmed og Dr. Wegdan Hasha kan du også kontaktes for ytterligere informasjon (469 07 322/455 31 756)

Hvem kan delta i studien?

- Migrantbakgrunn (fødested utenfor Norge) og lovlig opphold i Norge
- Høyere utdanning
- Ikke i relevant arbeid tross høyere utdanning
- Norsknivå A2-B1
- Bosatt i Kristiansand
- Ønsker å svare på spørreundersøkelsen igjen etter 6 måneder

Hva innebærer deltagelse?

Det eneste vi ønsker at du skal gjøre er å besvare spørreundersøkelsen to ganger i løpet av året. Som godtgjørelse for deltagelse får hver deltager et gavekort på 150 kr når andre spørreundersøkelse er gjennomført mai/juni 2023.

Rett til å trekke deg og nekte deltagelse

Deltagelse er frivillig. Du trenger ikke å delta hvis du ikke ønsker dette. Du kan trekke deg når som helst under studien uten å oppgi noen grunn. All informasjon om deg vil da bli slettet. Når prosjektet avsluttes 30.04.2027 anonymiseres gjenstående data.

Ditt personvern – hvordan vi oppbevarer og bruker dine opplysninger

Vi vil ikke dele din informasjon med andre. Det er bare forskerne i studien som har tilgang til informasjonen og uvedkommende har ikke innsyn. Navnet og kontaktopplysningene dine vil jeg erstatte med en kode som lagres på egen navneliste adskilt fra øvrige data.
Vi lagrer all informasjon på en sikker datamaskin.
Vi sletter lydopptak fra intervjuet når vi har skrevet ned alt som vi har snakket om.
Vi passer på at ingen kan kjenne deg igjen når vi skriver forskningsartikler.
Vi vil for eksempel finne opp et annet navn når vi skriver om deg.
Vi følger loven om personvern.

Dine rettigheter

Så lenge du kan identifiseres i datamaterialet, har du rett til:
· innsyn i hvilke opplysninger vi behandler om deg, og å få utlevert en kopi av opplysningene
· å få rettet opplysninger om deg som er feil eller misvisende
· å få slettet personopplysninger om deg
· å sende klage til Datatilsynet om behandlingen av dine personopplysninger

Hvis du har spørsmål knyttet til Personverntjenester sin vurdering av prosjektet, kan du ta kontakt med: · Personverntjenester på epost (personverntjenester@sikt.no) eller på telefon: 53 21 15 00.

Hva gir oss rett til å behandle personopplysninger om deg?

Vi behandler opplysninger om deg basert på ditt samtykke. På oppdrag fra Universitetet i Bergen har Personverntjenester vurdert at behandlingen av personopplysninger i dette prosjektet er i samsvar med personvernregelverket.

Samtykke for å gjennomføre spørreundersøkelsen:

Jeg har lest informasjonen over, eller så har jeg fått den opplest. Jeg har hatt muligheten til å stille spørsmål, og spørsmål som jeg har hatt, har blitt besvart. Jeg velger å delta frivillig for å gjennomføre spørreundersøkelsene og delta i studien. Jeg vet at jeg kan trekke meg fra deltagelsen når som helst, uten å måtte gi begrunnelse. Jeg vet også at jeg kan få innsyn i mine data.

Dato:

Deltager sin signatur   Forskningsansvarlig sin signatur

Consent and invitation letter to participate in the study:

“Health through meaningful integrative occupational activities for highly educated immigrants - a pilot intervention study”

Background information: purpose of the study

The University of Bergen is conducting a pilot intervention study that will enable highly educated migrants with a health professional background that has not been approved of in Norway, to participate in teaching activities at UiB and HVL. The pilotstudy is a part of a PhD-thesis.

The intervention will measure the effect of meaningful integration on health among migrants and their families. Our understanding of the term meaningful integration entails that the participants will use their own expertise as much as possible. Furthermore, we also want to investigate how the presence of migrants at educational institutions contributes to increased cultural awareness and cultural competence among staff and students.

The results of the research will contribute to an increased knowledge base about meaningful integration among highly educated migrants in Norway. This is an invitation letter to participate in the study and a consent form to answer in the questionnaires twice.

Who oversees the research project?

Project manager is Dr.Esperanza Diaz. PhD-student Khadra Yasien Ahmed and Dr. Wegdan Hasha can also be contacted for further information 469 07 322/455 31 756

Who can participate in the study?

- Migrant background (place of birth outside Norway) and legal residence in Norway

- Higher education (minimum high school)
- Not in relevant work despite higher education
- Norwegian level A2-B1
- Resident of Kristiansand municipality
- Would like to answer the survey again after 6 months

What does participation entail for you?

The only thing we want you to do is answer the survey twice. As a compensation for your time, we will give you a gift card valued 150 NOK once the second survey is cunducted in may/june.

Right to withdraw and refuse participation

Participation is voluntary. You don't have to participate if you don't want to. You can withdraw at any time during the study without giving any reason. All information about you will then be deleted. When the research project ends on 30.04.2027, all data will be anonymized.

Your privacy - how we store and use your information

We will not share your information with others. Only the researchers in the study have access to the information and unauthorized have no access. We will replace your name and contact details with a code that is stored on a separate name list separate from other data.
We store all information on a secure computer.
We delete audio recordings from the interview when we have written down everything we have talked about.
We make sure that no one can recognize you when we write research articles. For example, we will invent a different name when we write about you.
We adhere to the laws on privacy.

Your rights

If information about you appears in what we write, or have in our documents, you have the right to see what information about you we collect. You can also request that the information be deleted so that it no longer exists. If there is any information that is incorrect, you can report it and ask the researcher to correct it. You can also ask to receive a copy of the information from us. You can also complain to the Norwegian Data Protection Authority if you think that we have processed the information about you in a careless way or in a way that is not correct.

If you have questions related to the Data protections' assessment of the project, you can contact this service at: personverntjenester@sikt.no or 53 21 15 00

What gives us the right to process your personal data?

We process information about you based on your consent. On behalf of the University of Bergen, Personal Protection Services has assessed that the processing of personal data in this project is in accordance with the privacy regulations.

Consent for participation in the study:

I have read the information, or it has been read to me. I have had the opportunity to ask questions about it and any questions that I have asked, have been answered. I consent voluntarily to conduct the questionaries twice and partake in the study. I know that I can refuse to participate at any time without having to explain why.

Date:

Participant's signature Researcher's signature

UNIVERSITY OF BERGEN

Invitasjon til deltagelse som mentor i en studie:

"Helse gjennom meningsfulle integrerende yrkesaktiviteter for høyt utdannede innvandrere - en pilotintervensjonsstudie"

Bakgrunnsinformasjon

Universitetet i Bergen inviterer deg til å delta som mentor i en intervensjonsstudie som skal tilrettelegge for at høyt utdannede migranter med helsefaglig bakgrunn som ikke er godkjent i Norge, kan delta i utdanningsrettede aktiviteter ved UiB og HVL.

Intervensjonen skal måle effekten av meningsfull integrering på helse blant migranter. Resultatene av forskningen skal bidra til økt kunnskapsgrunnlag om meningsfull integrering blant høyt utdannede migranter i Norge. Dette er et informasjonsskriv om rollen som mentor for en seksmåneders periode og et samtykkeskjema for å delta som mentor og bli intervjuet i etterkant av intervensjonen.

Deltageren vil være under veiledning av faggruppen de blir innlemmet i og samtidig ha en del selvstendig ansvar. Hvor mye ansvar den enkelte skal få, avtales mellom deg og migranten som skal utplasseres hos dere. Vi vil undersøke hvordan denne prosessen har vært, Ph.d.-kandidaten vil intervjue mentorene samt observere hvordan samspillet mellom mentorene og migrantene er.

Hvem er ansvarlig for forskningsprosjektet?

Prosjektleder Dr. Esperanza Diaz og Phd-student Khadra Yasien Ahmed (46907322) som du også kan kontakte for ytterliger informasjon

Hvem søker vi?

- Faglig ansatte ved HVL eller UIB
- Villig til å være hovedkontaktperson for en eller flere migranter under utplassering
- Åpen for veiledning og inkludering av migranter i eget arbeid
- Ønske om å delta i seks måneder, gjerne lenger

Intervensjonen har følgende steg for mentorer

-Matche mentor og fagmiljø med rett deltager

-Gjennomføre veiledning med migrantene

-Evaluering ved hjelp av to intervjuer

Rett til å trekke deg og nekte deltagelse

Deltagelse er frivillig. Du trenger ikke å delta hvis du ikke ønsker dette. Du kan trekke deg når som helst under studien uten å oppgi noen grunn. All informasjon om deg vil da bli slettet. Vi oppbevarer dine data forsvarlig og i henhold til personvernloven.

Så lenge du kan identifiseres i datamaterialet, har du rett til:

· innsyn i hvilke opplysninger vi behandler om deg, og å få utlevert en kopi av opplysning

å få rettet opplysninger om deg som er feil eller misvisende

· å få slettet personopplysninger om deg

· å sende klage til Datatilsynet om behandlingen av dine personopplysninger

All data oppbevares forsvarlig på en sikker datamaskin og anonymiseres etter prosjektets utløpsdato, 30.04.2027.

Ditt personvern – hvordan vi oppbevarer og bruker dine opplysninger

Vi vil ikke dele din informasjon med andre. Det er bare forskerne i studien som har tilgang til informasjonen og uvedkommende har ikke innsyn. Navnet og kontaktopplysningene dine vil jeg erstatte med en kode som lagres på egen navneliste adskilt fra øvrige data.

Vi lagrer all informasjon på en sikker datamaskin.

Vi sletter lydopptak fra intervjuet når vi har skrevet ned alt som vi har snakket om.

Vi passer på at ingen kan kjenne deg igjen når vi skriver forskningsartikler. Vi vil for eksempel finne opp et annet navn når vi skriver om deg.

Intervjuet vil bli tatt opp, transkribert og analysert. Intervjuene skal anonymiseres, lagres konfidensielt uten navn, men knyttes til en kode.

Hvis du har spørsmål knyttet til Personverntjenester sin vurdering av prosjektet, kan du ta kontakt med: Personverntjenester på epost (personverntjenester@sikt.no) eller på telefon: 53 21 15 00.

Hva gir oss rett til å behandle personopplysninger om deg?

Vi behandler opplysninger om deg basert på ditt samtykke. På oppdrag fra Universitetet i Bergen har Personverntjenester vurdert at behandlingen av personopplysninger i dette prosjektet er i samsvar med personvernregelverket.

Samtykke for å stille som mentor

Jeg har lest informasjonen over, eller så har jeg fått den opplest. Jeg har hatt muligheten til å stille spørsmål, og spørsmål som jeg har hatt, har blitt besvart. Jeg velger å delta frivillig i studien og stille til intervju. Jeg vet at jeg kan trekke meg fra deltagelsen når som helst, uten å måtte gi begrunnelse. Jeg vet også at jeg kan få innsyn i mine data.

Dato:

Mentor sin signatur **Forskningansvarlig sin signatur:**

UNIVERSITY OF BERGEN

Invitation to participate as a mentor in a study

"Health through meaningful integrative occupational activities for highly educated immigrants - a pilot intervention study"

Background information

The University of Bergen invites you to partake in a pilot intervention study as a mentor. This pilot study will enable highly educated migrants with a health professional background that has not been approved of in Norway, to participate in teaching activities at UiB and HVL. The pilot study is a part of an PhD-thesis.

The intervention will measure the effect of meaningful integration on health among migrants and their families. Our understanding of the term meaningful integration entails that the participants will use their own expertise as much as possible. Furthermore, we also want to investigate how the presence of migrants at educational institutions contributes to increased cultural awareness and cultural competence among staff and students.

The results of the research will contribute to an increased knowledge base about meaningful integration among highly educated migrants in Norway. This is an invitation and consent letter to participate in the study as a mentor and be interviewed.

Who oversees the research project?

Project manager: Dr. *Esperanza Diaz* and PhD student *Khadra Yasien Ahmed (46907322)* that you also can contact for further information

Who are we looking for and what does participation entail?

- Academic staff at HVL or UIB
- Willing to be the main contact person for one or more migrants during deployment
- Willing to instruct and include migrants in their own work
- Desire to participate/commit for six months, preferably longer

The intervention has the following steps for mentors:

- Obtain consent forms from mentors to partake in the study
- Matching mentor and the professional group with the right participants
- Final interviews with mentors

Right to withdraw and refuse participation

Participation is voluntary. You don't have to participate if you don't want to. You can withdraw at any time during the study without giving any reason. All information about you will then be deleted. We store your data properly and in accordance with the Personal Protection Act.

As long as you can be identified in the data material, you have the right to:

· access to the information we process about you, and to be given a copy of the information

· to have information about you corrected that is incorrect or misleading

· to have personal data about you deleted

· to send a complaint to the Norwegian Data Protection Authority about the processing of your personal data

All data is stored securely on a secure computer and anonymized after the project's expiry date, 30.04.2027

If you have questions related to the Data protections' assessment of the project, you can contact this service at: personverntjenester@sikt.no or 53 21 15 00

What gives us the right to process your personal data?

We process information about you based on your consent. On behalf of the University of Bergen, Personal Protection Services has assessed that the processing of personal data in this project is in accordance with the privacy regulations.

Consent for participation in the study:

I have read the information, or it has been read to me. I have had the opportunity to ask questions about it and any questions that I have asked, have been answered. I consent voluntarily to partake in the study and be interviewed. I know that I can refuse to participate at any time without having to explain why.

Date:

Mentor's signature **Researchers signatur**

Date:

Place:

Health through meaningful integrative occupational activities for highly educated migrants – a pilot intervention study

Thank you for taking part in this study!

The information in this questionnaire will be used in research aimed at understanding the experience of health among migrants with higher health-related education in Norway. It is important that you answer all the questions. Ask if there is something you don't understand. The completed questionnaire must be returned to the people responsible for the study.

Please answer by putting an X in the box (☐), or answer the open fields ( ) as explained in the text.

By answering this survey, you agree that we use the information only for the purpose of the study. All information will be treated confidentially.

The Integration for health-group,
The University of Bergen

1. PERSONALIA

1.1 First name:

Please specify

1.2 Last name:

Please specify

1.3 Phone:

Please specify

1.4 Email address:

Please specify

2. BACKGROUND INFORMATION

2.1 Gender:

☐ Male ☐ Female ☐ Other

2.2 Date of birth:

__ . __ . ____ *(e.g. 01.06.1978)*

2.3 Country of birth:

Please specify

2.4 Year of arrival to Norway: ____ *(e.g. 2020)*

2.5 Reason for emigrating to Norway:

☐ Work ☐ Refuge/asylum ☐ Family reunification

☐ Studies ☐ Other

2.6 Tick the box that best describes your level of proficiency in Norwegian:

A1	A2	B1	B2	C1	C2
☐	☐	☐	☐	☐	☐

2.7 Marital status:

- ☐ Married ☐ Divorced ☐ Widow/widower
- ☐ Single ☐ Cohabiting ☐ In a relationship

2.8 How many children do you have?

No children	1	2	3	4	5	6 or more
☐	☐	☐	☐	☐	☐	☐

2.9 How many people, including yourself, live in your household?

1	2	3	4	5	6 or more
☐	☐	☐	☐	☐	☐

2.10 Municipality of residence:

Please specify

3. EDUCATION AND EMPLOYMENT

3.1 In what field did you work before you arrived in Norway?
(You may select more than one option)

- ☐ I did not work ☐ Military occupations
- ☐ Leadership ☐ Academic occupations
- ☐ Office work ☐ University college occupations
- ☐ Farming/fishing ☐ Sales and service occupations
- ☐ Craftship ☐ Transportation
- ☐ Cleaning ☐ Process and machine operators
- ☐ Helper ☐ Other

3.2 Which of the following options best describes what you have been doing for the last four weeks?
(Please select only one)

- ☐ Paid employment
- ☐ Helper/assistant
- ☐ Unpaid labour (household chores, childcare, or similar)
- ☐ Unemployed, actively looking for a job
- ☐ Unemployed, not actively looking for a job
- ☐ Permanently sick or disabled
- ☐ Retired
- ☐ In school
- ☐ Introduction program/qualification program
- ☐ Other

3.3 In what field is your educational background?

- ☐ Natural sciences and technical subjects
- ☐ Health sciences (medicine, nursing, pharmacy, or similar)
- ☐ Social sciences, humanities or law
- ☐ Vocational specialization
- ☐ Pedagogy
- ☐ IT/media
- ☐ Other

3.4 If you have a health professional background, please specify your health professional background:

Please specify

3.5 How many years of schooling have you completed after high school?
(Including higher education)

☐☐ *(e.g. 10)*

3.6 Which country/countries is your education from?

Please specify

3.7 What degree can your education level be categorized as in Norway?

- ☐ Bachelor's degree ☐ Master's degree
- ☐ Doctorate (Ph.D.) ☐ Vocational education
- ☐ None of the above

	Yes	No
3.8 Do you have any incomplete or interrupted degrees/studies?	☐	☐

3.9 If yes, within which field?

- ☐ Natural sciences and technical subjects
- ☐ Health sciences (medicine, nursing, pharmacy, or similar)
- ☐ Social sciences, humanities or law
- ☐ Vocational specialization
- ☐ Pedagogy
- ☐ IT/media
- ☐ Other

	Yes	No
3.10 Is your educational background relevant to your current job? ***(E.g. nursing for someone employed within health care)***	☐	☐

	Yes	No	No need
3.11 Have you applied for approval of your education in Norway?	☐	☐	☐

3.12 If yes: How much of your education did you have to redo/repeat?

☐ Nothing ☐ The entire education
☐ Some subjects ☐ Almost the entire education
☐ Don't know

4. HEALTH, FUNCTION LEVEL AND QUALITY OF LIFE

4.1 How do you consider your health at the moment?

Very good	Good	Neither	Poor	Very poor
☐	☐	☐	☐	☐

	Yes	No
4.2 Do you suffer from long-term (at least 1 year) illness or injury of a physical or psychological nature that impairs your daily life?	☐	☐

4.3 If yes, how would you describe your impairment?

	Slight	Moderate	Severe
4.3.1 Motor ability impairment	☐	☐	☐
4.3.2 Vision impairment	☐	☐	☐
4.3.3 Hearing impairment	☐	☐	☐
4.3.4 Impairment due to physical illness	☐	☐	☐
4.3.5 Impairment due to mental health problems	☐	☐	☐

4.4 How many hours do you approximately sit during a normal day?
(Including both work hours and leisure time)

About ☐ hours *(e.g. 6 hours)*

5. WELL-BEING

5.1 Please respond to each of the statements below by marking the option that best describes how you have felt during <u>the last two weeks</u>:

	All of the time					Never
5.1.1 I have felt cheerful and in good spirits	5	4	3	2	1	0
5.1.2 I have felt calm and relaxed	5	4	3	2	1	0
5.1.3 I have felt active and vigorous	5	4	3	2	1	0
5.1.4 I have woken up feeling fresh and rested	5	4	3	2	1	0
5.1.5 My daily life has been filled with things that interest me	5	4	3	2	1	0

6. GENERAL HEALTH QUESTIONNAIRE

6.1 During <u>the past two weeks</u>, have you:

6.1.1 Been able to concentrate on what you have been doing?	Better than usual	As usual	Less than usual	A lot less than usual
6.1.2 Lost much sleep over worry?	Has not happened	Not more than usual	More than usual	I slept a lot less than usual
6.1.3 Felt that you are playing a useful part in things?	More than usual	As usual	Less than usual	A lot less than usual
6.1.4 Felt capable of making decisions about things?	More than usual	As usual	Less than usual	A lot less than usual

6.1.5 Felt constantly under strain?	Not at all	Not more than usual	More than usual	A lot more than usual
6.1.6 Felt you couldn't overcome your difficulties?	Not at all	Not more than usual	More than usual	A lot more than usual
6.1.7 Been able to enjoy your normal day to day activites?	More than usual	As usual	Less than usual	A lot less than usual
6.1.8 Been able to face up to your problems?	Better than usual	As usual	Less than usual	A lot less than usual
6.1.9 Been losing confidence in yourself?	Not at all	Not more than usual	More than usual	A lot more than usual
6.1.10 Been feeling unhappy or depressed?	Not at all	Not more than usual	More than usual	A lot more than usual
6.1.11 Been thinking about yourself as a worthless person?	Not at all	Not more than usual	More than usual	A lot more than usual
6.1.12 Been feeling reasonably happy for day-to-day activities?	More than usual	As usual	Less than usual	A lot less than usual

7. INTEGRATION

7.1 How connected do you feel with Norway?

- ☐ I feel an extremely close connection
- ☐ I feel a very close connection
- ☐ I feel a moderately close connection
- ☐ I feel a weak connection
- ☐ I do not feel a connection at all

7.2 How often do you feel like an outsider in Norway?

Never	Rarely	Some times	Often	Always
☐	☐	☐	☐	☐

7.3 When you think about your future, where do you want to live?

- ☐ I will definitely live in Norway for the rest of my life
- ☐ I will probably live in Norway for the rest of my life
- ☐ I am not sure whether I want to stay in Norway or move to another country
- ☐ I will probably move to another country
- ☐ I will definitely move to another country

7.4 How often do you feel isolated from the Norwegian society?

Never	Rarely	Some times	Often	Always
☐	☐	☐	☐	☐

7.5 How difficult or easy would it be for you to find a job in Norway?

- ☐ Very difficult
- ☐ Somewhat difficult
- ☐ Neither difficult nor easy
- ☐ Somewhat easy
- ☐ Very easy

7.6 In the last 12 months, how often did you eat dinner with Norwegians who are not part of your family?

- ☐ Never
- ☐ Once a year
- ☐ Once a month
- ☐ Once a week
- ☐ Almost every day

7.7 Please think about the Norwegians in your phone contacts or among your social media contacts. With how many of them did you have a conversation - either by phone, messenger chat, or text exchange, in the last 4 weeks?

☐ 0 ☐ 1–2 ☐ 3–6

☐ 7–14 ☐ 15 or more

7.8 Please evaluate your own skills in Norwegian. How well can you do the following when reading Norwegian? I can read and understand the main points in simple newspaper articles on familiar subjects:

- ☐ Very well
- ☐ Well
- ☐ Moderately well
- ☐ Not well
- ☐ Not well at all

7.9 Please evaluate your own skills in Norwegian. How well can you do the following when speaking Norwegian? In a conversation, I can speak about familiar topics and express personal opinions:

- ☐ Very well
- ☐ Well
- ☐ Moderately well
- ☐ Not well
- ☐ Not well at all

8. DISCRIMINATION

8.1 How often do you experience the following situations in Norway?

	Never	Seldom	Some times	Most of the time	Often	Very often
8.1.1 Discrimination by Norwegian authorities	1	2	3	4	5	6
8.1.2 Discrimination in school or at work	1	2	3	4	5	6
8.1.3 Feeling disrespected due to your national background	1	2	3	4	5	6
8.1.4 People making racist remarks towards you	1	2	3	4	5	6

9. MENTAL HEALTH

9.1 Please mark the option that best describes how much each of the following feelings has bothered you during the last week.

	Not at all	A little	Somewhat	Very much
9.1.1 Suddenly scared for no reason	1	2	3	4
9.1.2 Felt scared	1	2	3	4
9.1.3 Fainting, dizziness or weakness	1	2	3	4
9.1.4 Felt tense or tired	1	2	3	4
9.1.5 Blamed yourself for things	1	2	3	4
9.1.6 Difficulty falling or staying asleep	1	2	3	4
9.1.7 Felt sad	1	2	3	4
9.1.8 Felt worthless	1	2	3	4
9.1.9 Felt that everything is an effort	1	2	3	4
9.1.10 Felt hopeless about the future	1	2	3	4

10. SENSE OF COHERENCE

10.1 Here is a series of questions relating to various aspects of your life. Each question has seven possible answers. Please mark the number the best expresses your answer.
Please give only one answer to each question

	Very seldom or never						Very often
10.1.1 Do you have the feeling that you don't really care about what goes on around you?	1	2	3	4	5	6	7

	Never happened						Always happened
10.1.2 Has it happened in the past that you were surprised by the behaviour of people you thought you knew well?	1	2	3	4	5	6	7

	Never happened						Always happened
10.1.3 Has it happened that people whom you counted on disappointed you?	1	2	3	4	5	6	7

	1	2	3	4	5	6	7
	No clear goals or purpose at all						Very clear goals and purpose
10.1.4 Until now your life has had:	1	2	3	4	5	6	7
	Very often						Very seldom or never
10.1.5 Do you have the feeling that you're being treated unfairly?	1	2	3	4	5	6	7
	Very often						Very seldom or never
10.1.6 Do you have the feeling that you are in an unfamiliar situation and don't know what to do?	1	2	3	4	5	6	7
	A source of deep pleasure and satisfaction						A source of pain and boredom
10.1.7 Doing the thing you do every day is:	1	2	3	4	5	6	7
	Very often						Very seldom or never
10.1.8 Do you have very mixed-up feelings and ideas?	1	2	3	4	5	6	7
	Very often						Very seldom or never
10.1.9 Does it happen that you have feelings inside you would rather not feel?	1	2	3	4	5	6	7
	Never						Very often
10.1.10 Many people – even those with a strong character – sometimes feel like sad sacks (losers) in certain situations. How often have you felt this way in the past?	1	2	3	4	5	6	7
	You overestimated or underestimated its importance						You saw things in the right proportion
10.1.11 When something happened, have you generally found that:	1	2	3	4	5	6	7
	Very often						Very seldom or never
10.1.12 How often do you have the feeling that there's little meaning in the things you do in your daily life?	1	2	3	4	5	6	7
	Very often						Very seldom
10.1.13 How often do you have feelings that you're not sure you can keep under control?	1	2	3	4	5	6	7

11. FOLLOW-UP

	Yes	No
11.1 Finally, we would like to ask you for your permission to contact you again for the project after six months to conduct a new survey. It is important for us to know how you are doing in terms of health.	☐	☐

THANK YOU FOR ANSWERING THESE QUESTIONS! PLEASE MAKE SURE TO RETURN THIS FORM TO THE PERSON WHO GAVE IT TO YOU BEFORE LEAVING.

Midtveisevaluering/Midway evaluation

-Observere interaksjon, språkbruk, arbeidsfordeling av oppgaver og generell integrering

Spørsmål til underveisevaluering for deltagere

Hensikten med intervjuet er å lære mer om hvordan deltakerne opplever intervensjonen midtveis for å gjøre nødvendige endringer i tide for å forbedre resultatet

1. Hva har du gjort så langt i intervensjonen?
2. Hvordan føler kursdagene har forberedt deg for utplassering?
3. Hvordan har planleggingen med mentor gått? Hvordan har du bidratt direkte og indirekte?
4. Hvordan er det ved instituttet/faggruppen du er tilknyttet?
5. Hvordan har det vært å undervise på norsk?
6. Hvordan er det å delta på undervisning av norske studenter?
7. Hvordan har intervensjonen påvirket din og din families helse så langt?
8. Hvilke utfordringer har oppstått og hvordan har du løst dem?

Med mentor

Med instituttet/faggruppen

Questions to the midway- evaluation for participants

The purpose of the interview is to learn more about how the participants are experiencing the intervention midway to make the necessary changes as to improve the end-result

Observe interaction, language use, tasks preformed, division of tasks and general integration.

1. What have you done so far in the intervention?
2. In what way did the two course days prepare you for the placement at UIB-HVL?
3. How was the planning the teaching with the mentor? How have you contributed directly and indirectly?
4. How is it at the department / research group you are affiliated with?
5. How has it been to teach in Norwegian?
6. What is it like to participate in teaching Norwegian students?
7. How has the intervention affected your and your family's health so far?

8. What challenges have arisen and how have you solved them? With mentor

With the department / research group/institute

Midterm Evaluation Questions for Mentors Purpose:
The aim of this interview is to understand how mentors experience the intervention at its midpoint, in order to make timely adjustments that can improve the final outcomes.

Observation Focus:
Interaction, language use, task performance, division of responsibilities, and overall integration.

1. **How has the intervention been so far?**
   (Describe your involvement and responsibilities.)
2. **How did you experience planning and collaboration with the participant?**
   - In what ways has the participant contributed directly and indirectly?
3. **How would you describe the participant's integration into the department/research group?**
   (Socially, professionally, etc.)
4. **How has the participant managed teaching or other research related activities ?***(What support have they needed or received?)*
5. **Have you noticed any effects of the intervention on the participant's well-being or family situation?**
   (If applicable and appropriate to comment on.)
6. **What challenges have you encountered during the intervention, and how have you addressed them?**
7. **What suggestions do you have for improving the second half of the intervention?** *(Support, structure, communication and how can the research group help YOU for the remaining time)*

UNIVERSITY OF BERGEN

Dybdeintervju- guide for deltagere

Helse gjennom meningsfulle integrerende yrkesaktiviteter for høyt utdannede innvandrere - en pilotintervensjonsstudie

Deltagelse i intervensjonen

- Hvilket utbytte sitter du igjen med etter deltagelse i intervensjonen?
- Hvor mye selvstendig arbeid utførte du?
- Hvor mye samarbeid du med andre?
- På hvilken måte fikk du brukt din egen faglige erfaring/kompetanse?
- Hvordan gikk kommunikasjonen med studentene?
- Hvis du fikk undervise eller delta i forskningsaktiviteter, hvordan opplevde du dette?
- Hva var utfordringen med å være deltager i intervensjonen?
- - Hva fungerte(bra)?

Effekt på helse

Tror du det er noen sammenheng mellom helsen din og deltagelse i intervensjonen?

-på hvilken måte i så fall?

Tror du at intervensjonen har påvirket din familie sin helse, og i så fall hvordan?

Opplevd effekt

Hvordan tror du din tilstedeværelse ved UIB/HVL har påvirket fagmiljøet/instituttet?

Hvordan tror du din tilstedeværelse ved ditt institutt/fakultet har påvirket studentene?

Hvordan tror du din tilstedeværelse ved instituttet/faggruppen har påvirket seg selv?

Avslutning:

-Hva som kan gjøres bedre?

- Noen siste innspill?

Takk for deltakelse!

In-depth interview guide for participants

Health through meaningful integrative occupational activities for highly educated immigrants - a

pilot intervention study

Participation in the intervention

What benefit are you left with after participating in the intervention?

How much independent work did you do?

How much did you collaborate with others?

In what way did you get to use your own professional

expertise/experience? In what way did you get to use your intercultural

competence?

How did the communication with the students go?

If you were invoved in any teaching or reserach related tasks: what experiences are you left with?

What was the challenge of being a participant in the intervention?

- What worked (well)?

Effect on health

Do you think there is any connection between your health and participation in the intervention?
-if so, in what way?

Do you think the intervention has affected your family's health, and if so how?

Perceived effect

How do you think your presence at HVL/UIB has affected the research group/department?

How do you think your presence at your department/faculty has affected the students?

How do you think your presence at the UIB/HVL has affected yourself?

-What can be done better?

- Any last input? Thank you for participating

Intervju-guide til mentorer

Helse gjennom meningsfulle integrerende yrkesaktiviteter for høyt utdannede innvandrere - en pilotintervensjonsstudie

Mentorrollen

Hvordan har det vært å ha deltagere ved deres faggruppe?

Hvordan opplevde du personlig å ha med deg en deltager?

Hvilke typer arbeidsoppgaver ble utført?

Hva er utfordringen med å være mentor?

- Hva fungerte/hva var bra?

Opplevd effekt

Hvordan tror du deltagernes tilstedeværelse har påvirket fagmiljøet ved din faggruppe?

Hvordan tror du deltagernes tilstedeværelse ved fagmiljøet har påvirket studentene?

Hvordan tror du deltagernes tilstedeværelse ved faggruppe/institutt har påvirket deg?

Hvordan tror du deltagernes tilstedeværelse ved faggruppe/institutt har påvirket deltageren selv?

Avslutning:

-Hvordan kan denne mentorordningen gjøres bedre?

- Noen siste innspill?

- Takk for deltakelse!

Interview guide for mentors

"Health through meaningful integrative occupational activities for highly educated immigrants - a pilot intervention study"

Assessment of the mentor role:

-How did you experience having participants at your institute/research group?

- How did you personally experience having a participant with you?

- What job tasks did you carry out?

-What was the challenge of being a mentor?

- What worked?

Perceived effect

-How do you think the participants' presence at the faculty has affected the students?

How do you think the participants' presence at the faculty has affected your research group?

How do you think the participants' presence at the faculty has affected you?

How do you think the participants' presence at the faculty has affected the participants personally?

Lastly:

-Any thoughts on how to improve this program?

- Any last inputs?

Thank you for participating!

Follow-up av deltagere ett år etter intervensjonen:

Hei

Som et ledd i oppfølgningen av deltagerne i intervensjon-studien, ønsker vi nå 12 måneder etter oppstart å ha en oppfølgingssamtale over telefonen med deltagerne.

Denne samtalen skal ikke tas opp eller transkriberes, men jeg vil notere meg noen momenter. Spørsmålene stilles ut ifra et forhåndsbestemte skjema dette er for å sikre at alle blir stilt de samme spørsmålene. Spørsmålene vil ta for seg hva de bedriver av aktiviteter om dagen, hvorvidt de er i jobb og hvordan de tror deres nåværende jobb/praksissituasjon kan ha blitt påvirket av deltagelse i studien.

Hver deltager vil bli intervjuet kun en gang mellom 10-15 minutter og vi vil innhente deres samtykke muntlig om hvorvidt de ønsker å stille til en slik samtale. Deltagerne vil få informasjon i forkant og avtale tid som passer dem.

Å få innsikt i hva deltagerne gjør etter at studien er avsluttet er av stor verdi da vi kan undersøke nærmere om deltagelse i studien var til nytte eller ei for deltagerne.

Takk.

Dette er spørsmålene til deltagerne:

1. Hvordan har du hatt det siden sist?
2. Hva gjør du om dagen?
3. Hvis jobb: hvor ?
4. Hvis NAV/o.l; type praksis?
5. Søk av godkjennelse av utdanning; før/under/etter studien?
6. Hvordan tror du deltagelse i intervensjonen har påvirket din nåværende situasjon (jobb/praksis o.l)
7. Hvis forverring/forbedring i helse, hvordan tror du dette har blitt påvirket av intervensjonen?
8. Noen siste tanker?

Follow-up with Participants One Year After the Intervention

Hello,
As part of the follow-up of participants in the intervention study, we would now like to conduct a follow-up phone interview with participants, 12 months after the start of the study.

This conversation will not be recorded or transcribed, but I will take notes on a few key points. The questions will be asked based on a pre-determined questionnaire to ensure that all participants are asked the same questions. The questions will focus on what activities the participants are currently engaged in, whether they are employed, and how they believe their current job or internship situation may have been influenced by their participation in the study.

Each participant will be interviewed only once, for approximately 10–15 minutes, and we will obtain their verbal consent regarding whether they wish to participate in such a conversation. Participants will receive information in advance and schedule a time that suits them.

Gaining insight into what participants are doing after the study has concluded is of great value, as it allows us to further investigate whether participation in the study was beneficial for them.

Thank you.

Questions for Participants:

1. How have you been since we last spoke?
2. What are you currently doing?
3. If employed: where?
4. If receiving support (e.g., from NAV): what type of internship/practice?
5. Have you applied for recognition of your education: before/during/after the study?
6. How do you think participation in the intervention has affected your current situation (job/internship, etc.)?
7. If your health has improved or worsened, how do you think this has been influenced by the intervention?
8. Any final thoughts?
