# Supplementary material for: Changes in health after a work-related intervention among highly educated migrants in Norway: a pilot study
Source: BMC Public Health. 2025 Oct 31;25:3699. doi: 10.1186/s12889-025-25025-9 (PMC12577434; doi:10.1186/s12889-025-25025-9)
Supplement: Supplementary file 2 — Supplementary Material 2. [file 12889_2025_25025_MOESM2_ESM.pdf]

Dato:

Sted:

Helse gjennom meningsfulle integrerende yrkesrettede aktiviteter – En pilot intervensjonsstudie

Takk for at du deltar i denne studien!

Informasjonen i dette spørreskjemaet vil bli brukt i forskning rettet mot å forstå opplevelse av helse blant migranter med høyere helseutdanning i Norge. Det er viktig at du besvarer alle spørsmålene. Spør hvis det er noe du ikke forstår. Det utfylte spørreskjemaet skal gis tilbake til personene som inviterte deg til studien.

Svar ved å markere boksen (☐) eller besvare de åpne feltene ( ) som forklart i teksten.

Ved å besvare denne spørreundersøkelsen samtykker du til at vi bruker informasjonen kun til studiens formål. All informasjon behandles konfidensielt.

Hilsen Integrering for helse-gruppen,
Universitetet i Bergen

1. PERSONALIA

1.1 **Fornavn:**

Vennligst spesifiser

1.2 **Etternavn:**

Vennligst spesifiser

1.3 **Telefonnummer:**

Vennligst spesifiser

1.4 **Epostadresse:**

Vennligst spesifiser

2. BAKGRUNNSINFORMASJON

2.1 **Kjønn:**

☐ Mann ☐ Kvinne ☐ Annet

2.2 **Fødselsdato:**

__ . __ . ____ *(f.eks. 01.06.1978)*

2.3 **Fødeland:**

Vennligst spesifiser

2.4 **Ankomstår til Norge:** ____ *(f.eks. 2020)*

2.5 **Årsak til innvandring til Norge:**

☐ Arbeid ☐ Flukt/asyl ☐ Familiegjenforening
☐ Studier ☐ Annet

2.6 **Kryss av boksen som best beskriver ditt nivå av norskkunnskaper:**

A1	A2	B1	B2	C1	C2
☐	☐	☐	☐	☐	☐

2.7 Sivilstand:

- [ ] Gift
- [ ] Skilt
- [ ] Enke/enkemann
- [ ] Singel
- [ ] Samboende
- [ ] I et forhold

2.8 Hvor mange barn har du?

Ingen barn	1	2	3	4	5	6 eller flere
☐	☐	☐	☐	☐	☐	☐

2.9 Hvor mange er dere totalt i husstanden, inkludert deg selv?

1	2	3	4	5	6 eller flere
☐	☐	☐	☐	☐	☐

2.10 Bostedskommune:

Vennligst spesifiser

3. UTDANNING OG ARBEID

3.1 Hva jobbet du med før du kom til Norge?

(Du kan velge flere alternativer)

- [ ] Uten arbeid
- [ ] Militære yrker
- [ ] Lederyrker
- [ ] Akademiske yrker
- [ ] Kontoryrker
- [ ] Høyskoleyrker
- [ ] Bonde/fisker
- [ ] Salg- og serviceyrker
- [ ] Håndtverker
- [ ] Transportarbeider
- [ ] Renhold
- [ ] Prosess- og maskinoperatør
- [ ] Hjelpearbeider
- [ ] Annet

3.2 Hvilket alternativ beskriver best det du har gjort <u>de siste fire ukene</u>?

(Velg kun ett alternativ)

- [ ] Lønnet arbeid
- [ ] Hjelpearbeider/assistent
- [ ] Ulønnet arbeid (husarbeid, barnepass, e.l.)
- [ ] Arbeidsledig, aktivt jobbsøkende
- [ ] Arbeidsledig, ikke aktivt jobbsøkende
- [ ] Varig syk eller funksjonshemmet
- [ ] Pensjonist
- [ ] Skole
- [ ] Introduksjonsprogrammet/kvalifiseringsprogrammet
- [ ] Annet

3.3 Hva er din utdanningsbakgrunn?

- [ ] Tekniske og naturvitenskapelige fag
- [ ] Helsefag (medisin, sykepleie, farmasi e.l.)
- [ ] Samfunnsvitenskap, humaniora eller jus
- [ ] Yrkesfaglig retning
- [ ] Pedagogikk
- [ ] IT/media
- [ ] Annet

3.4 Hvis du har helsefaglig utdannelse, vennligst oppgi hva slags helseutdanning du har:

Vennligst spesifiser

3.5 Hvor mange år med skolegang har du fullført etter videregående skole?

(Inkludert høyere utdannelse)

(f.eks. 10)

3.6 Hvilke(t) land er utdannelsen din fra?

Vennligst spesifiser

3.7 Hvilken grad kan utdannelsen din kategoriseres under i Norge?

- [ ] Bachelorgrad
- [ ] Mastergrad
- [ ] Doktorgrad
- [ ] Yrkesfaglig utdanning
- [ ] Ingen av disse

3.8 Har du en utdannelse du ikke har fullført, eller som har blitt avbrutt?

Ja	Nei
☐	☐

3.9 Hvis ja, innen hvilket fagområde?

- [ ] Tekniske og naturvitenskapelige fag
- [ ] Helsefag (medisin, sykepleie, farmasi e.l.)
- [ ] Samfunnsvitenskap, humaniora eller jus
- [ ] Yrkesfaglig retning
- [ ] Pedagogikk
- [ ] IT/media
- [ ] Annet

3.10 Er utdanningen din relevant for jobben din nå?
(F.eks. sykepleie for en person som jobber i helsevesenet)

Ja	Nei
☐	☐

3.11 Har du søkt om å få utdannelsen din godkjent i Norge?

Ja	Nei	Trenger ikke
☐	☐	☐

3.12 Hvis ja, hvor mye av utdannelsen må du ta på nytt?

☐ Ingen deler
☐ Hele utdanningen
☐ Kun noen fag
☐ Nesten hele utdanningen
☐ Vet ikke

4. HELSE, LIVSKVALITET OG FUNKSJON

4.1 Hvordan vurderer du alt i alt din egen helse?

Svært god	God	Verken god eller dårlig	Dårlig	Svært dårlig
☐	☐	☐	☐	☐

4.2 Lider du av langvarig (minst 1 års) sykdom eller skade av fysisk eller psykisk art som svekker ditt daglige liv?

Ja	Nei
☐	☐

4.3 Hvis ja, hvordan vil du beskrive svekkelsen?

	Lett	Moderat	Alvorlig
4.3.1 Motorisk svekkelse	☐	☐	☐
4.3.2 Nedsatt syn	☐	☐	☐
4.3.3 Nedsatt hørsel	☐	☐	☐
4.3.4 Svekkelse på grunn av fysisk sykdom	☐	☐	☐
4.3.5 Svekkelse på grunn av psykiske helseproblemer	☐	☐	☐

4.4 Hvor mange timer av dagen er du stillesittende?
(Både hjemme, på skolen og på jobb)

Omtrent ☐☐ timer *(f.eks. 6 timer)*

5. TRIVSEL OG VELVÆRE

4.1 Merk alternativet som best beskriver hvordan du har følt deg i løpet av <u>de siste to ukene</u>:
(Høyere tall betyr bedre velvære)

	Hele tiden					Aldri
5.1.1 Jeg har følt meg glad og i godt humør	5	4	3	2	1	0
5.1.2 Jeg har følt meg rolig og avslappet	5	4	3	2	1	0
5.1.3 Jeg har følt meg aktiv og sterk	5	4	3	2	1	0
5.1.4 Jeg har følt meg opplagt og uthvilt når jeg våkner	5	4	3	2	1	0
5.1.5 Jeg har følt at mitt daglige liv har vært fylt av ting som interesserer meg	5	4	3	2	1	0

6. GENERELLE HELSESPØRSMÅL

6.1 I løpet av <u>de siste to ukene</u>, har du:

6.1.1 Vært i stand til å konsentrere deg om det du har drevet med?	Mer enn vanlig	Samme som vanlig	Mindre enn vanlig	Mye mindre enn vanlig
6.1.2 Mistet søvn på grunn av bekymringer?	Ikke i det hele tatt	Ikke mer enn vanlig	Heller mer enn vanlig	Mye mer enn vanlig
6.1.3 Følt at du tar del i ting på en nyttig måte?	Mer enn vanlig	Samme som vanlig	Mindre enn vanlig	Mye mindre enn vanlig
6.1.4 Følt at du er i stand til å ta beslutninger om ulike ting?	Mer enn vanlig	Samme som vanlig	Mindre enn vanlig	Mye mindre enn vanlig

6.1.5 Følt deg stadig under press?	Ikke i det hele tatt	Ikke mer enn vanlig	Heller mer enn vanlig	Mye mer enn vanlig
6.1.6 Følt deg ute av stand til å mestre vanskeligheter?	Ikke i det hele tatt	Ikke mer enn vanlig	Heller mer enn vanlig	Mye mer enn vanlig
6.1.7 Vært i stand til å glede deg over dine daglige gjøremål?	Mer enn vanlig	Samme som vanlig	Mindre enn vanlig	Mye mindre enn vanlig
6.1.8 Vært i stand til å møte utfordringer?	Mer enn vanlig	Samme som vanlig	Mindre enn vanlig	Mye mindre enn vanlig
6.1.9 Mistet troen på deg selv?	Ikke i det hele tatt	Ikke mer enn vanlig	Heller mer enn vanlig	Mye mer enn vanlig
6.1.10 Følt deg ulykkelig eller deprimert?	Ikke i det hele tatt	Ikke mer enn vanlig	Heller mer enn vanlig	Mye mer enn vanlig
6.1.11 Tenkt på deg selv som en verdiløs person?	Ikke i det hele tatt	Ikke mer enn vanlig	Heller mer enn vanlig	Mye mer enn vanlig
6.1.12 Stort sett følt deg bra i hverdagen	Mer enn vanlig	Samme som vanlig	Mindre enn vanlig	Mye mindre enn vanlig

7. INTEGRERING

7.1 Hvor knyttet føler du deg til Norge?

- ☐ Jeg fløer en ekstremt nær forbindelse
- ☐ Jeg føler en veldig nær forbindelse
- ☐ Jeg føler en moderat tilknytning
- ☐ Jeg føler en svak tilknytning
- ☐ Jeg føler ikke en tilknytningi det hele tatt

7.2 Hvor ofte kjenner du på utenforskap i Norge?

Aldri	Sjelden	Noen ganger	Ofte	Alltid
☐	☐	☐	☐	☐

7.3 Når du tenker på fremtiden din, hvor vil du bo?

- ☐ Jeg vil definitivt bo i Norge resten av livet
- ☐ Jeg vil nok bo i Norge resten av livet
- ☐ Jeg er usikker på om jeg vil bli i Norge eller om jeg vil flytte til et annet land
- ☐ Jeg vil nok flytte til et annet land
- ☐ Jeg vil definitivt flytte til et annet land

7.4 Hvor ofte føler du deg isolert fra det norske samfunnet?

Aldri	Sjelden	Noen ganger	Ofte	Alltid
☐	☐	☐	☐	☐

7.5 Hvor vanskelig eller enkelt ville det være for deg finne jobb i Norge?

- ☐ Veldig vanskelig
- ☐ Litt vanskelig
- ☐ Verken vanskelig eller lett
- ☐ Litt lett
- ☐ Veldig lett

7.6 I løpet av de siste 12 månedene, hvor ofte har du spist med nordmenn som ikke er en del av din familie?

- ☐ Aldri
- ☐ En gang i året
- ☐ En gang i måneden
- ☐ En gang i uken
- ☐ Nesten hver dag

7.7 Tenk på nordmennene i telefonkontaktene dine eller blant kontaktene dine på sosiale medier. Hvor mange av dem hadde du en samtale med, enten via telefon, Messenger-chat eller tekstutveksling, i løpet av de siste 4 ukene?

☐ 0 ☐ 1–2 ☐ 3–6

☐ 7–14 ☐ 15 eller mer

7.8 Vennligst vurder dine egne ferdigheter i norsk. Hvor godt kan du gjøre følgende når du leser norsk? Jeg kan lese og forstå hovedpoengene i enkle avisartikler om kjente emner

- ☐ Veldig godt
- ☐ Godt
- ☐ Verken godt eller dårlig
- ☐ Dårlig
- ☐ Veldig dårlig

7.9 Vennligst vurder dine egne ferdigheter i norsk. Hvor godt kan du gjøre følgende når du snakker norsk? I en samtale kan jeg snakke om kjente temaer og uttrykke personlige meninger

- ☐ Veldig godt
- ☐ Godt
- ☐ Verken godt eller dårlig
- ☐ Dårlig
- ☐ Veldig dårlig

8. DISKRIMINERING

8.1 **Hvor ofte opplever du følgende situasjoner i Norge?**

	Aldri	Sjelden	Noen ganger	For det meste	Ofte	Veldig ofte
8.1.1 Diskriminering fra norske myndigheter	1	2	3	4	5	6
8.1.2 Diskriminering i skolen eller på jobben	1	2	3	4	5	6
8.1.3 Føler deg lite respektert på grunn av din nasjonale bakgrunn	1	2	3	4	5	6
8.1.4 Folk som kommer med rasistiske ytringer mot deg	1	2	3	4	5	6

9. PSYKISK HELSE

9.1 **Angi hvor mye du har vært plaget av de følgende symptomene i løpet av <u>den siste uken</u>.**

	Ikke plaget i det hele tatt	Litt plaget	Ganske plaget	Veldig plaget
9.1.1 Plutselig frykt uten grunn	1	2	3	4
9.1.2 Følt deg redd	1	2	3	4
9.1.3 Besvimelse, svimmelhet eller svakhet	1	2	3	4
9.1.4 Følt deg anspent eller sliten	1	2	3	4
9.1.5 Klandret deg selv for ting	1	2	3	4
9.1.6 Vanskeligheter med å sovne	1	2	3	4
9.1.7 Følt deg trist	1	2	3	4
9.1.8 Følt deg verdiløs	1	2	3	4
9.1.9 Følt at alt er et slit	1	2	3	4
9.1.10 Følt på håpløshet med hensyn til fremtiden	1	2	3	4

10. OPPLEVELSE AV SAMMENHENG

10.1 **Her er en rekke spørsmål knyttet til ulike aspekter av livet ditt. Marker tallet som best uttrykker ditt svar.** ***(Kun ett svar per spørsmål)***

	Veldig sjelden eller aldri						Veldig ofte
10.1.1 Føler du i bunn og grunn at du ikke bryr deg om hva som skjer rundt deg?	1	2	3	4	5	6	7

	Aldri						Alltid
10.1.2 Har det hendt at du ble overrasket over oppførselen til personer som du trodde du kjente godt?	1	2	3	4	5	6	7

	Aldri						Alltid
10.1.3 Har det hendt at du ble skuffet av personer som du har stolt på?	1	2	3	4	5	6	7

	Ingen klare mål eller hensikt						Svært klare mål og hensikt
10.1.4 Inntil nå har livet ditt hatt:	1	2	3	4	5	6	7

	Veldig ofte						Veldig sjelden eller aldri
10.1.5 Har du følt at du blir urettferdig behandlet?	1	2	3	4	5	6	7

	Veldig ofte						Veldig sjelden eller aldri
10.1.6 Har du opplevd å være i en ukjent situasjon der du ikke vet hva du skal gjøre?	1	2	3	4	5	6	7

	En kilde til dyp glede og tilfredshet						En kilde til smerte og kjedsomhet
10.1.7 Å gjøre det du gjør hver dag er:	1	2	3	4	5	6	7

	Veldig ofte						Veldig sjelden eller aldri
10.1.8 Har du veldig uklare følelser og tanker?	1	2	3	4	5	6	7

	Veldig ofte						Veldig sjelden eller aldri
10.1.9 Hender det at du har følelser inni deg som du ikke ønsker å ha?	1	2	3	4	5	6	7

	Aldri						Veldig ofte
10.1.10 Mange mennesker, selv karaktersterke, føler seg noen ganger som tapere i visse situasjoner. Hvor ofte har du følt det slik?	1	2	3	4	5	6	7

	Du over- eller undervurderte betydningen						Du så ting i riktig proporsjon
10.1.11 Når noe har hendt, har du generelt opplevd at:	1	2	3	4	5	6	7

	Veldig ofte						Veldig sjelden eller aldri
10.1.12 Hvor ofte føler du at det er liten mening i de tingene du gjør daglig?	1	2	3	4	5	6	7

	Veldig ofte						Veldig sjelden
10.1.13 Hvor ofte har du følelser som du ikke er sikker på at du kan holde under kontroll?	1	2	3	4	5	6	7

11. OPPFØLGING

	Ja	Nei
**11.1 Til slutt vil vi vite om du samtykker til å bli kontaktet igjen i forbindelse med studien etter seks måneder for å gjennomføre en ny spørreundersøkelse. Det er viktig for oss å vite hvordan det går med din helse.**	☐	☐

TAKK FOR AT DU SVARER PÅ DISSE SPØRSMÅLENE! HUSK Å RETURNERE DETTE SKJEMAET TIL PERSONEN SOM GAV DEG DET FØR DU DRAR.
