# Supplementary material for: Changes in health after a work-related intervention among highly educated migrants in Norway: a pilot study
Source: BMC Public Health. 2025 Oct 31;25:3699. doi: 10.1186/s12889-025-25025-9 (PMC12577434; doi:10.1186/s12889-025-25025-9)
Supplement: Supplementary file 3 — Supplementary Material 3. [file 12889_2025_25025_MOESM3_ESM.docx]

**Additional table 3:** *An overview what data from which group and time of measurement (N=123).*

| **Group** | **Participants** | **Time point** | **Collected data** |
| --- | --- | --- | --- |
|  |  |  |  |
| **Intervention** | 21 recruited | Baseline | Questionnaire (quantitative) |
|  | 19 allocated |  |  |
|  | 15 completed | Follow-up (6 months) | Questionnaire (quantitative) + Interviews (qualitative) |
|  | 4 lost to follow-up | Follow-up (12 months) | Phone interviews (qualitative notes only) |
|  |  |  |  |
| **Control** | 102 recruited | Baseline | Questionnaire (quantitative) |
|  | 62 completed | Follow-up (6 months) | Questionnaire (quantitative) |

**Additional table 4:** *An overview of the themes and sorted codes identified.    (N=19)*

| **Theme** | **Codes / Key Concepts** | **Example Quotes** |
| --- | --- | --- |
| Renewed Self-Confidence | Professional reintegration, language improvement, empowerment, pride, identity | “Just being able to be present in a workplace… it gives you confidence and empowerment.” – Thabita  “I did so many tasks, it gave me more confidence in myself.” – Safiya |
| Professional Reintegration | Joys and ambiguities, cognitive dissonance, validation, hope, reassurance | “It was a huge accomplishment… I felt like I can use my expertise and my abilities.” – Hilal  “Now I feel I'm a bit more a somebody. Although I was kind of comfortable with being nobody.” – Ali |
| Family Wellbeing and Pride | Children’s perception, family dynamics, emotional support, confirmation/acknowledgement/pride | “My children say, ‘Mum has a job’… Not like before when I used to be sitting at home.” – Kawther  “My children have also been very proud… They say my mother works in the university.” – Yamina |
| Mental Health and Stress Management | Reduced stress, meaningful activity, coping strategies | “Mentally, I wasn't feeling okay… But I know how to handle it now.” – Hawa  “My stress lowered.” – Rashid |
| Navigating the System | System knowledge, mentor support, planning next move | “Now I have a plan. I'm sure many others do too.” – Maryam  “They helped me a lot to get to know the easiest ways to get my authorization.” – Fatima |
| Physical Activity and Routine | Increased movement, daily structure, gender differences, women, new routines | “Physically, I was out every day… Before I used to eat at home, and I didn’t know what I was going to do.” – Fatima  “In terms of the physical, I am a lot more active.” – Amal |

**Additional table 5:** *An overview of education and sex distribution of mentees and mentors (n=26)*


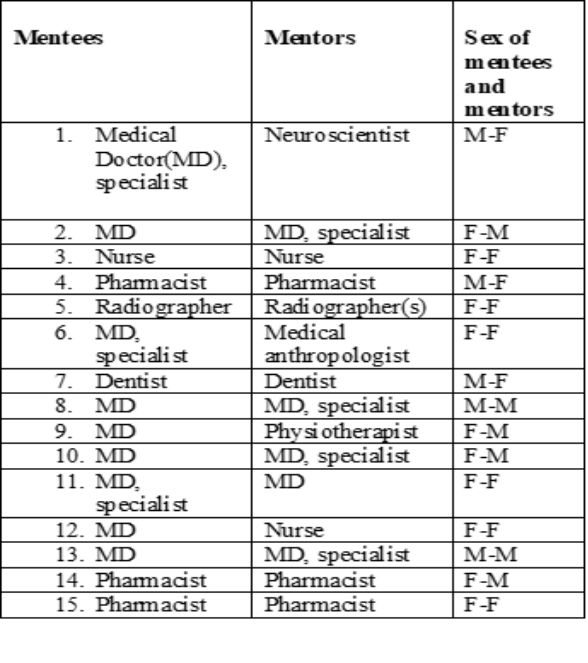


|  | **ADDITIONAL TABLE 6:** *OVERVEIW OF MISSING VS NOT MISSING* | | |
| --- | --- | --- | --- |
|  | **Not missing follow-up** | **Missing follow-up   data** | **p-value** |
| N= | 77.0 (62.6%) | 46.0 (37.4%) |  |
|  |  |  |  |
| Age (median, IQR) | 30.0 (34.0-41.0) | 28.0 (33.0-39.0) | 0.773 |
|  |  |  |  |
| Gender |  |  |  |
| Women | 45 (58.4%) | 29 (63.0%) | 0.614 |
|  |  |  |  |
| YEAR  of ARRIVAL |  |  |  |
| 2016-2020 | 33 (42.9%) | 13 (28.3%) | **0.105** |
| 2021-2023 | 44 (57.1%) | 33 (71.7%) |  |
| NUMBER of children |  |  |  |
| 0 | 33 (42.9%) | 20 (43.5%) | **0.083** |
| 1 | 15 (19.5%) | 16 (34.8%) |  |
| 2+ | 29 (37.7%) | 10 (21.7%) |  |
|  |  |  |  |
| cg-KRISTIANSAND | 62 (80.5%) | 40 (87.0%) | 0.359 |
| ig-Bergen | 15 (19.5%) | 6 (13.0%) |  |
