# Supplementary material for: Changes in health after a work-related intervention among highly educated migrants in Norway: a pilot study
Source: BMC Public Health. 2025 Oct 31;25:3699. doi: 10.1186/s12889-025-25025-9 (PMC12577434; doi:10.1186/s12889-025-25025-9)
Supplement: Supplementary file 4 — Supplementary Material 4. [file 12889_2025_25025_MOESM4_ESM.docx]

| **Outcomes at baseline and follow-up in intervention and control groups** | | | | **Effect in intervention versus control groups** | | |
| --- | --- | --- | --- | --- | --- | --- |
|  | **n (%)** | **n (%)** | **n (%)** | **n (%)** | **Crude OR**^1^ *  **(95% CI)** | **Adjusted OR** ^1,3^  **(95% CI)** |
| SRH | -1.29  (0.74) | -2.08  (0.99) | -2.99  (0.73) | -2.12  (0.61) | 0.21 **^1^**  (0.02; 2.18) | 0.19  (0.02, 2.00) |
|  | **Mean (SD)** | **Mean (SD)** | **Mean (SD)** | **Mean (SD)** | **Crude difference**^2^  **(95% CI)** | **Adjusted difference** ^3^ **(95% CI)** |
| GHQ-12 | 0.48 (0.01) | 0.45 (0.01) | 0.44 (0.01) | 0.49  (0.01) | -0.07  (-0.11; -0.02) | -0.07  (-0.11; 0.02) |
| HSCL-10 | 0.29 (0.04) | 0.25  (0.03) | 0.27 (0.02) | 0.21  (0.02) | 0.03  (-0.07; 0.12) | 0.02  (-0.07; 0.11) |
| WHO-5 | 0.68 (0.04) | 0.78 (0.03) | 0.68  (0.02) | 0.69  (0.02) | 0.09  (0.01; 0.17) | 0.09  (0.01; 0.17) |
| SOC-13 | 0.55 (0.02) | 0.55 (0.02) | 0.55 (0.01) | 0.55  (0.01) | 0.01  (-0.07; 0.08) | 0.00  (-0.07; 0.08) |
| ILP- 12 | 0.56 (0.21) | 0.65 (0.23) | 0.62 (0.01) | 0.67  (0.01) | 0.04 (-0.02; 0.10) | 0.041 (-0.02; 0.01) |
| Discrimination | 0.22 (0.04) | 0.15 (0.04) | 0.10 (0.01) | 0.13  (0.01) | -0.10 (-0.21; 0.02) | -0.10  (-0.21; 0.02) |

**^1^** Ratio of prevalence ratio (RPR): prevalence ratio in the intervention group divided by the prevalence ratio in the control group (reference).

^2^ Difference: differences in differences in means.

^3^ Adjusted for gender, age, year since arrival and number of children.

*The SRH here is on the odds-scale, the example in the point-by-point is not. Just to illustrate the non-significant change across scales.
